# Regional Anaesthesia Training in a United Kingdom Postgraduate Anaesthesia Programme: A Cross-Sectional Survey of Training Opportunities, Supervision and Competency Development

**DOI:** 10.7759/cureus.117719

**Published:** 2026-10-10

**Authors:** Jakub Kazda

**Affiliations:** 1 Anaesthetics, Hull University Teaching Hospitals National Health Service (NHS) Trust, Hull, GBR

**Keywords:** anaesthesiology, clinical competence, graduate medical education (gme), questionnaires and surveys, regional anaesthesiology, residency and internship

## Abstract

Background

Regional anaesthesia is an integral component of contemporary anaesthetic practice and a core competency within the 2021 Royal College of Anaesthetists curriculum. Despite increasing emphasis on regional anaesthesia training, concerns remain regarding trainees' access to sufficient clinical exposure and supportive clinical supervision.

Objective

This study described regional anaesthesia training experiences reported by anaesthesia trainees within a United Kingdom (UK) postgraduate training programme, including training opportunities, procedural exposure, self-reported curriculum attainment and barriers to competency development.

Methods

A cross-sectional electronic survey (questionnaire) was distributed to all anaesthesia trainees within the Yorkshire and Humber School of Anaesthesia between January and February 2024. The questionnaire explored access to educational resources, procedural exposure, self-reported attainment of curriculum competencies, perceived training adequacy, and perceived barriers to training. Descriptive analyses were performed, with secondary exploratory analyses examining differences in perceived training adequacy according to respondent characteristics and reported training experiences.

Results

Sixty-four trainees completed the survey (estimated response rate: 25.6%). Twenty respondents (31.2%) reported sufficient opportunities to achieve curriculum-mandated regional anaesthesia competencies, while 25 (39.1%) reported insufficient opportunities. The most frequently reported barriers were lack of supportive supervising anaesthetists (60.9%) and limited access to suitable operating lists (59.4%). In exploratory comparisons, respondents reporting adequate access to clinical regional anaesthesia practice had higher perceived training adequacy scores, while respondents reporting limited access to suitable operating lists or lack of supportive supervision had lower scores. Reported access to educational resources and perceived training adequacy also varied among respondents from different training hubs. Stage 3 respondents reported variable procedural exposure across individual regional anaesthesia techniques and variable self-reported ability across anatomical regions.

Conclusions

This regional survey identified variation in reported regional anaesthesia training experiences among respondents from a single UK postgraduate training programme. Exploratory comparisons identified differences in perceived training adequacy according to reported clinical opportunities, suitable operating lists, and supportive clinical supervision; these findings are hypothesis-generating and warrant prospective evaluation. Stage 3 respondents reported variable procedural exposure and self-reported ability, neither of which should be regarded as a surrogate marker of objectively assessed competence. The findings support continued emphasis on competency-based assessment and further evaluation of supervised clinical training and consistency of curriculum delivery.

## Introduction

Regional anaesthesia has become an integral component of contemporary anaesthetic practice. Ultrasound-guided peripheral nerve blocks and fascial plane blocks provide effective perioperative analgesia, reduce opioid requirements, improve patient satisfaction, and support enhanced recovery pathways across a wide range of surgical procedures [1]. Therefore, competence in regional anaesthesia is now considered a core skill for modern consultant anaesthetists.

In the United Kingdom, the 2021 Royal College of Anaesthetists (RCoA) Curriculum integrates regional anaesthesia throughout postgraduate anaesthetic training, with trainees expected to develop competence in an increasing range of techniques as they progress through training. These requirements closely align with the Regional Anaesthesia UK (RA-UK) Plan A blocks, a group of high-value, evidence-based techniques considered essential for routine anaesthetic practice [2].

Delivering high-quality regional anaesthesia training remains challenging. Increasing service demands, workforce pressures, reduced training opportunities, and variation in local expertise can limit access to supervised clinical experience [3,4]. Recent national surveys have demonstrated considerable variation in the provision of regional anaesthesia training across the United Kingdom, including inconsistent access to advanced techniques, dedicated teaching, and experienced supervisors [5,6]. As competency-based training increasingly replaces procedural targets, understanding trainees' educational experiences and perceived barriers to learning has become increasingly important.

Although previous studies have described regional and national provision of regional anaesthesia training, relatively few have explored trainees' perceptions of curriculum delivery within individual training programmes. Trainees' perceptions of the educational environment provide valuable insight into curriculum implementation and have been associated with learner motivation, engagement, self-efficacy and clinical performance, while also identifying variation in trainees' educational experiences [7].

The objective of this study was therefore to describe the RA training experiences reported by respondents from a single UK postgraduate anaesthesia programme, including access to educational resources and clinical opportunities, procedural exposure, self-reported curriculum attainment, perceived training adequacy and perceived barriers to training. Secondary exploratory analyses examined associations between perceived training adequacy and selected respondent characteristics and reported training experiences.

## Materials and methods

Study design and setting

A cross-sectional questionnaire-based survey was conducted between January and February 2024 among anaesthesia trainees enrolled in the Yorkshire and Humber School of Anaesthesia, United Kingdom. The school comprises three regional training programmes based in Hull, Leeds, and Sheffield and delivers postgraduate anaesthesia training across all stages of the 2021 RCoA Curriculum. Reporting of this study follows the consensus-based Checklist for Reporting of Survey Studies (CROSS) [8].

Survey instrument

The questionnaire was developed with reference to the 2021 RCoA Curriculum requirements for regional anaesthesia and previously published literature describing barriers to procedural skills training [4-6]. It was adapted in part from the previously published United Kingdom (UK) national survey of regional anaesthesia training by Bellew et al. [5]. In particular, the assessment of procedural exposure across individual regional anaesthesia techniques and self-reported ability across anatomical regions was based on domains assessed in the national survey. Additional questions were developed to examine curriculum attainment, access to educational resources and perceived barriers within the regional training programme. The questionnaire was reviewed by a regional expert in regional anaesthesia education before it was disseminated. The adapted instrument did not undergo separate formal pilot testing, cognitive interviewing, psychometric validation, or assessment of test-retest reliability or internal consistency.

The questionnaire contained eight questions presented to all respondents, followed by stage-specific questions determined by the respondent's training stage. Stage 1 and Stage 2 trainees completed two additional questions, while Stage 3 trainees completed four additional questions. Responses consisted of Likert-scale, multiple-choice and categorical items. The questionnaire was administered using Google Forms (Google LLC; Mountain View, CA, USA). The complete questionnaire is provided as appendices.

Participants and data collection

All anaesthesia trainees within the Yorkshire and Humber School of Anaesthesia were eligible to participate, irrespective of stage of training. Approximately 250 trainees were enrolled across the three regional training programmes at the time of survey distribution, based on contemporaneous regional recruitment numbers. All eligible trainees were invited to participate, representing a census approach rather than sampling of a predetermined proportion of the population. No a priori sample-size calculation was performed, and the achieved sample size was determined by voluntary survey participation.

The questionnaire was distributed once electronically through the regional trainee mailing lists and educational networks; no reminder invitations were sent. Participation was voluntary and anonymous, and no incentives were offered. No technical mechanism was used to prevent multiple submissions. All questions presented to each respondent were mandatory; consequently, there were no missing data for applicable questions and no imputation was required. Submission of a completed questionnaire was considered to imply informed consent to participate.

Hub-specific eligible trainee numbers were unavailable; therefore, hub-specific response rates could not be calculated. No information was collected from non-responders, and no weighting or statistical adjustment for non-response was performed.

Study outcomes

The primary outcome was trainees' perceived adequacy of opportunities to achieve curriculum-mandated regional anaesthesia competencies, measured using a five-point Likert scale ranging from strongly disagree to strongly agree.

Secondary outcomes included self-reported access to educational resources, attainment of curriculum competencies, procedural exposure, ability to perform selected regional anaesthesia techniques and perceived barriers to training.

For trainees undertaking Stage 3 training, procedural exposure was recorded separately for individual regional anaesthesia techniques using predefined categories (0, 1-5, 6-10, 11-20, 21-30, and >30 procedures). These data were analysed descriptively without applying a procedural threshold for competence. The published expert consensus recommends adequate practical experience as part of competency development in regional anaesthesia [9]; however, because this recommendation relates to experience within anatomical regions rather than individual block techniques, it was not applied as a threshold to the technique-specific exposure data collected in this survey. Self-reported ability was assessed separately for regional anaesthesia of the upper limb, lower limb, chest wall, and abdominal wall.

Statistical analysis

No prospectively registered or prespecified statistical analysis plan was used. Data were analysed using descriptive and inferential statistics. Descriptive analyses were undertaken to characterise the survey responses. Secondary inferential analyses were exploratory and were undertaken to examine associations rather than to test prespecified hypotheses. Given the modest sample size and small numbers within several subgroups, p-values and effect sizes from these analyses should be interpreted cautiously.

Categorical variables are presented as frequencies and percentages, while ordinal variables are reported as medians with interquartile ranges (IQR).

Comparisons of perceived training adequacy between training stages and regional training programmes were performed using the Kruskal-Wallis test. Perceived training adequacy was compared according to access to individual educational resources and between respondents who did and did not report individual barriers using the Mann-Whitney U test. Cliff's delta was calculated as a measure of effect size for binary between-group comparisons.

Differences in access to educational resources between training hubs were assessed using Pearson's chi-square test of independence. Expected cell counts were examined, and where expected counts were <5, the Fisher-Freeman-Halton exact test was used for the corresponding 3 × 2 contingency table. To compare perceived training adequacy according to access to the five educational resources, p-values were adjusted for multiple comparisons using the Holm method.

All statistical tests were two-sided, with p < 0.05 used as the threshold for statistical significance in these exploratory analyses.

Statistical analyses were performed using Python version 3.13.5 (Wilmington, DE, USA) with SciPy version 1.17.0. Data were visualised using Matplotlib version 3.10.8.

Governance and ethics

This project was undertaken as an educational quality improvement and service evaluation initiative. No identifiable personal information was collected, and all responses were anonymous. Approval to distribute the survey was obtained from the Yorkshire and Humber School of Anaesthesia. In accordance with local governance guidance, formal research ethics committee approval was not required.

## Results

Respondent characteristics

A total of 64 anaesthesia trainees completed the survey, representing an estimated response rate of 25.6% based on approximately 250 trainees enrolled across the Yorkshire and Humber School of Anaesthesia at the time of survey distribution.

All three regional training hubs were represented, including Leeds (n = 25, 39.1%), Hull (n = 22, 34.4%) and Sheffield (n = 17, 26.6%). Respondents represented all stages of postgraduate training, with 24 (37.5%) Stage 1 trainees, 18 (28.1%) Stage 2 trainees and 20 (31.3%) Stage 3 trainees. Two respondents (3.1%) had not yet completed a regional anaesthesia module. Participant characteristics are summarised in Table 1.

**Table 1:** Characteristics of Survey Respondents (n = 64)

Characteristic	n	%
Training hub		
Leeds	25	39.1
Hull	22	34.4
Sheffield	17	26.6
Training stage		
Stage 1	24	37.5
Stage 2	18	28.1
Stage 3	20	31.3
No completed regional anaesthesia module	2	3.1
Estimated overall response rate	64/250	25.6

Perceived adequacy of regional anaesthesia training

Twenty respondents (31.2%; 95% confidence interval (CI): 21.2%-43.4%) agreed or strongly agreed that they had sufficient opportunities to achieve the regional anaesthesia competencies expected for their current stage of training. Twenty-five respondents (39.1%; 95% CI: 28.1%-51.3%) disagreed or strongly disagreed, while 19 (29.7%; 95% CI: 19.9%-41.8%) expressed a neutral opinion. The overall perceived adequacy score was 3 (IQR: 2-4) on the five-point Likert scale.

Access to educational resources

Access to educational resources varied considerably between learning modalities (Table 2). Appropriate access to clinical regional anaesthesia practice was reported by 32 respondents (50.0%), while 30 (46.9%) considered formal teaching opportunities to be adequate and 27 (42.2%) reported satisfactory access to ultrasound workshops or supervised scanning sessions.

**Table 2:** Perceived Access to Regional Anaesthesia Educational Resources by Training Hub Values are presented as n (%). Differences in reported access to educational resources between training hubs were assessed using Pearson's chi-square test of independence. Where expected cell counts were < five, the Fisher-Freeman-Halton exact test was used. All tests were two-sided, and statistical significance was defined as p < 0.05.

Educational resource	Hull (n = 22)	Sheffield (n = 17)	Leeds (n = 25)	Statistical test/statistic	p-value
Formal teaching	5 (22.7)	12 (70.6)	13 (52.0)	χ² = 9.25	0.0098
Ultrasound scanning	2 (9.1)	11 (64.7)	14 (56.0)	χ² = 15.37	<0.001
Cadaveric training	0 (0.0)	2 (11.8)	12 (48.0)	Fisher-Freeman-Halton exact	<0.001
High-fidelity simulation	0 (0.0)	2 (11.8)	1 (4.0)	Fisher-Freeman-Halton exact	0.262
Clinical practice	7 (31.8)	11 (64.7)	14 (56.0)	χ² = 4.74	0.093

Advanced educational modalities were substantially less accessible. Fourteen trainees (21.9%) reported adequate access to cadaveric regional anaesthesia courses, whereas only three respondents (4.7%) reported access to high-fidelity regional anaesthesia simulation.

Access to formal teaching (χ² = 9.25, p = 0.0098), ultrasound workshops (χ² = 15.37, p < 0.001) and cadaveric training (Fisher-Freeman-Halton exact, p < 0.001) differed significantly between regional training hubs. Access to routine clinical practice and high-fidelity simulation did not differ significantly between hubs.

Barriers to clinical exposure

The most frequently reported barriers to regional anaesthesia training were lack of supportive supervising anaesthetists (n = 39, 60.9%) and limited access to suitable operating lists (n = 38, 59.4%). Surgeon reluctance to utilise regional anaesthesia was reported by 10 respondents (15.6%), while patient refusal was uncommon (n = 3, 4.7%). The frequency of reported barriers is summarised in Figure 1.

**Figure 1:**
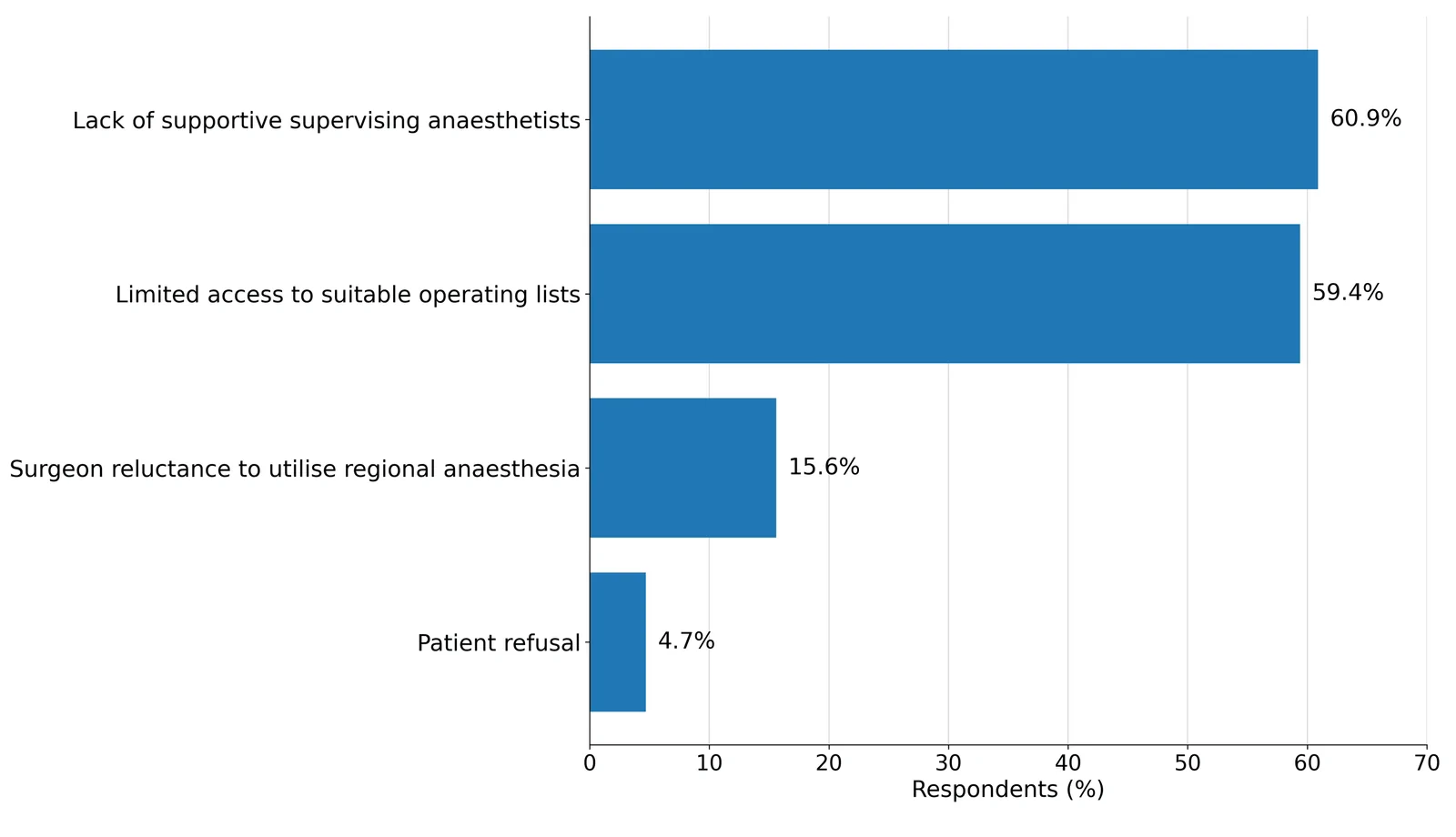
Reported Barriers to Clinical Regional Anaesthesia Training Among Survey Respondents The most frequently reported barriers to clinical regional anaesthesia training were lack of supportive supervising anaesthetists (60.9%) and limited access to suitable operating lists (59.4%). Surgeon reluctance to utilise regional anaesthesia (15.6%) and patient refusal (4.7%) were reported less frequently. Percentages are based on all survey respondents (n = 64). The figure was created using Python version 3.13.5 with Matplotlib version 3.10.8.

Attainment of curriculum outcomes

Attainment of curriculum outcomes varied across stages of training (Table 3).

**Table 3:** Self-Reported Attainment of Regional Anaesthesia Curriculum Competencies by Training Stage

Training stage/curriculum competency	Yes, n (%)	No, n (%)	Unsure, n (%)
Stage 1 (n = 24)			
Simple peripheral nerve block under local supervision	4 (16.7)	20 (83.3)	-
Fascia iliaca/femoral nerve block	10 (41.7)	6 (25.0)	8 (33.3)
Stage 2 (n = 18)			
Ultrasound-guided brachial plexus block with distant supervision	7 (38.9)	8 (44.4)	3 (16.7)
Truncal fascial plane block with distant supervision	6 (33.3)	9 (50.0)	3 (16.7)
Stage 3 (n = 20)			
Independent brachial plexus blocks	13 (65.0)	1 (5.0)	6 (30.0)
Independent lower limb blocks	19 (95.0)	0 (0.0)	1 (5.0)
Independent chest wall blocks	11 (55.0)	6 (30.0)	3 (15.0)
Independent abdominal wall blocks	12 (60.0)	2 (10.0)	6 (30.0)

Among Stage 1 trainees, only four of 24 respondents (16.7%) reported being able to perform a simple peripheral nerve block under local supervision, although 10 respondents (41.7%) reported being able to perform a fascia iliaca or femoral nerve block independently.

Among Stage 2 trainees, seven of 18 respondents (38.9%) reported being able to perform ultrasound-guided brachial plexus blocks with distant supervision, while six (33.3%) reported the ability for chest and abdominal wall fascial plane blocks.

Among Stage 3 trainees, 13 of 20 respondents (65.0%) reported being able to perform brachial plexus blocks independently, compared with 19 (95.0%) for lower-limb regional anaesthesia, 11 (55.0%) for chest wall blocks and 12 (60.0%) for abdominal wall blocks.

Procedural exposure among stage 3 trainees

Procedural exposure among Stage 3 trainees varied considerably between individual regional anaesthesia techniques (Table 4). Procedural frequencies are presented descriptively using the predefined survey categories and were not interpreted against a specific competency threshold.

**Table 4:** Procedural Exposure to Individual Regional Anaesthesia Techniques Among Stage 3 Trainees (n = 20) Values are presented as n (%). Procedural exposure was self-reported using predefined categorical ranges. Individual technique frequencies are presented descriptively and are not interpreted against a specific procedural threshold for competency. PECS: pectoralis nerve blocks

Anatomical region	Regional anaesthesia technique	0 procedures	1-5 procedures	6-10 procedures	11-20 procedures	21-30 procedures	>30 procedures
Upper limb	Interscalene	0 (0.0%)	5 (25.0%)	7 (35.0%)	7 (35.0%)	0 (0.0%)	1 (5.0%)
	Supraclavicular	0 (0.0%)	5 (25.0%)	7 (35.0%)	6 (30.0%)	0 (0.0%)	2 (10.0%)
	Infraclavicular	16 (80.0%)	2 (10.0%)	2 (10.0%)	0 (0.0%)	0 (0.0%)	0 (0.0%)
	Axillary	0 (0.0%)	1 (5.0%)	7 (35.0%)	4 (20.0%)	6 (30.0%)	2 (10.0%)
Lower limb	Fascia iliaca	0 (0.0%)	1 (5.0%)	5 (25.0%)	6 (30.0%)	1 (5.0%)	7 (35.0%)
	Adductor canal	0 (0.0%)	7 (35.0%)	3 (15.0%)	4 (20.0%)	3 (15.0%)	3 (15.0%)
	Popliteal sciatic	0 (0.0%)	2 (10.0%)	3 (15.0%)	8 (40.0%)	3 (15.0%)	4 (20.0%)
	Ankle	1 (5.0%)	11 (55.0%)	6 (30.0%)	2 (10.0%)	0 (0.0%)	0 (0.0%)
Chest wall	Thoracic paravertebral	10 (50.0%)	7 (35.0%)	2 (10.0%)	1 (5.0%)	0 (0.0%)	0 (0.0%)
	Erector spinae plane	4 (20.0%)	8 (40.0%)	5 (25.0%)	2 (10.0%)	1 (5.0%)	0 (0.0%)
	Serratus anterior plane	10 (50.0%)	5 (25.0%)	4 (20.0%)	1 (5.0%)	0 (0.0%)	0 (0.0%)
	PECS	11 (55.0%)	7 (35.0%)	1 (5.0%)	1 (5.0%)	0 (0.0%)	0 (0.0%)
Abdominal wall	Transversus abdominis plane	0 (0.0%)	5 (25.0%)	4 (20.0%)	7 (35.0%)	3 (15.0%)	1 (5.0%)
	Rectus sheath	8 (40.0%)	4 (20.0%)	3 (15.0%)	4 (20.0%)	0 (0.0%)	1 (5.0%)

Upper-Limb Blocks

Distribution of upper-limb procedural experience differed substantially between individual techniques. Axillary blocks had the greatest reported exposure, with eight of 20 trainees (40.0%) reporting more than 20 procedures. Exposure to interscalene and supraclavicular blocks was predominantly within the 1-20 procedure range. Infraclavicular block exposure was notably limited, with 16 trainees (80.0%) reporting that they had never performed the technique.

Lower-Limb Blocks

Lower-limb procedural exposure was generally greater, although it varied by technique. Fascia iliaca and popliteal sciatic blocks showed the greatest exposure, with eight (40.0%) and seven (35.0%) trainees, respectively, reporting more than 20 procedures. Exposure to adductor canal blocks was more widely distributed across the procedural categories. Ankle block experience was comparatively limited, with 11 trainees (55.0%) reporting 1-5 procedures and six (30.0%) reporting 6-10 procedures.

Truncal Blocks

Exposure to individual truncal techniques was generally more limited and heterogeneous. Ten trainees (50.0%) had never performed a thoracic paravertebral block, 10 (50.0%) had never performed a serratus anterior plane block, and 11 (55.0%) had never performed a pectoral nerve block. Erector spinae plane block experience was more widely distributed, although eight trainees (40.0%) reported only 1-5 procedures.

Transversus abdominis plane blocks had the greatest reported exposure among the abdominal wall techniques, with seven trainees (35.0%) reporting 11-20 procedures and three (15.0%) reporting 21-30 procedures. Eight trainees (40.0%) had never performed a rectus sheath block.

Exploratory analyses of perceived training adequacy

Exploratory analyses examined associations between respondent characteristics, reported educational opportunities, reported barriers and perceived training adequacy. These analyses were not prespecified and should be interpreted as hypothesis-generating given the modest sample size and small subgroup sizes.

Among respondents, perceived training adequacy differed between regional training hubs (Kruskal-Wallis H(2) = 15.94, p < 0.001), with median ratings of 4 (IQR: 3-4) in Sheffield, 3 (IQR: 2-4) in Leeds and 2 (IQR: 1-3) in Hull. Perceived adequacy did not differ significantly according to stage of training (H(3) = 0.96, p = 0.810).

Respondents reporting adequate access to clinical regional anaesthesia practice had higher perceived training adequacy scores than those reporting inadequate access (median 4 (IQR: 3-4) vs. 2 (IQR: 1-3); Mann-Whitney U = 870.0, p < 0.001; Cliff's δ = 0.70). Differences were also observed according to reported access to ultrasound workshops (U = 720.0, Holm-adjusted p = 0.009; Cliff's δ = 0.44) and formal teaching (U = 686.0, Holm-adjusted p = 0.046; Cliff's δ = 0.35). No statistically significant differences were observed according to reported access to cadaveric training or high-fidelity simulation.

Respondents reporting limited access to suitable operating lists had lower perceived training adequacy scores than those who did not report this barrier (Mann-Whitney U = 131.5, p < 0.001; Cliff's δ = −0.73). Respondents reporting lack of supportive supervision as a barrier also had lower perceived adequacy scores than those who did not (median 2 (IQR): 1-3) vs. 4 (IQR: 3-4); Mann-Whitney U = 215.0, p < 0.001; Cliff's δ = −0.56). A complete summary of the exploratory analyses is presented in Table 5.

**Table 5:** Exploratory Comparisons of Perceived Regional Anaesthesia Training Adequacy Values are presented as medians and interquartile ranges (IQR). These analyses were exploratory. Differences in perceived training adequacy between two groups were assessed using the Mann-Whitney U test, while comparisons across training hubs and training stages were assessed using the Kruskal-Wallis test. Effect sizes for Mann-Whitney U comparisons are reported as Cliff's delta (δ), with direction according to the group ordering shown; negative values for barriers indicate lower perceived training adequacy among respondents reporting the barrier. For comparisons according to reported access to the five educational resources, p-values were adjusted for multiple comparisons using the Holm method. All tests were two-sided, and statistical significance was defined as p < 0.05.

Comparison	Group summary	Statistical test	Statistic	p-value	Effect size
Training hub	Sheffield: median 4 (IQR 3-4); Leeds: median 3 (IQR 2-4); Hull: median 2 (IQR 1-3)	Kruskal-Wallis	H(2) = 15.94	<0.001	-
Training stage	No completed RA module: median 2.5 (IQR 2.2-2.8); Stage 1: median 3 (IQR 2-3); Stage 2: median 3 (IQR 2-4); Stage 3: median 3.5 (IQR 2-4)	Kruskal-Wallis	H(3) = 0.96	0.81	-
Adequate access to clinical RA practice	Adequate: median 4 (IQR 3-4); inadequate: median 2 (IQR 1-3)	Mann-Whitney U	U = 870.0	<0.001	δ = 0.70
Adequate access to ultrasound workshops	Adequate: median 4 (IQR 2.5-4); inadequate: median 3 (IQR 1-3)	Mann-Whitney U	U = 720.0	0.009	δ = 0.44
Adequate access to formal teaching	Adequate: median 3 (IQR 2.2-4); inadequate: median 2.5 (IQR 1-3)	Mann-Whitney U	U = 686.0	0.046	δ = 0.35
Adequate access to cadaveric training	Adequate: median 3 (IQR 2.2-4); inadequate: median 3 (IQR 2-4)	Mann-Whitney U	U = 396.0	0.448	δ = 0.13
Adequate access to high-fidelity simulation	Adequate: median 4 (IQR 3.5-4.5); inadequate: median 3 (IQR 2-4)	Mann-Whitney U	U = 141.0	0.22	δ = 0.54
Limited access to suitable operating lists	Barrier reported: median 2 (IQR 1-3); not reported: median 4 (IQR 3-4.8)	Mann-Whitney U	U = 131.5	<0.001	δ = −0.73
Lack of supportive supervision	Barrier reported: median 2 (IQR 1-3); not reported: median 4 (IQR 3-4)	Mann-Whitney U	U = 215.0	<0.001	δ = −0.56
Surgeon reluctance to utilise RA	Barrier reported: median 2.5 (IQR 1.2-3); not reported: median 3 (IQR 2-4)	Mann-Whitney U	U = 209.5	0.255	δ = −0.22
Patient refusal	Barrier reported: median 2 (IQR 1.5-2.5); not reported: median 3 (IQR 2-4)	Mann-Whitney U	U = 54.5	0.234	δ = −0.40

Only three respondents reported access to high-fidelity regional anaesthesia simulation, none of whom were Stage 3 trainees. The small number of respondents reporting simulation access limits interpretation of this comparison.

## Discussion

This regional survey provides a granular evaluation of anaesthesia trainees' experiences of regional anaesthesia training within a single postgraduate training programme. Three principal findings emerged. First, many respondents perceived that opportunities to achieve curriculum-mandated regional anaesthesia competencies were insufficient. Second, respondents reported variation in access to educational resources between training hubs despite a common regional curriculum. Third, in exploratory analyses, perceived training adequacy differed according to respondents' reported access to clinical regional anaesthesia practice, suitable operating lists, and supportive clinical supervision.

Clinical opportunities, supervision and perceived training adequacy

In exploratory comparisons, respondents reporting adequate access to clinical regional anaesthesia practice had higher perceived training adequacy scores, while respondents reporting limited access to suitable operating lists had lower scores; both comparisons had large effect sizes (Cliff's δ = 0.70 and −0.73, respectively). Respondents reporting lack of supportive supervision as a barrier also had lower perceived training adequacy scores, with a large effect size (Cliff's δ = −0.56), and lack of supportive supervising anaesthetists was the most commonly reported barrier to regional anaesthesia training. These exploratory findings identify the clinical learning environment, including access to appropriate cases and supervision, as an area for further investigation.

Effective clinical supervision depends on factors including the supervisory relationship and opportunities for trainee participation [10], with appropriate feedback forming an important component of supervisory practice [10,11]. Workforce shortages, service pressures and operating room efficiency may influence procedural opportunities, while supervisory practice and trainee participation may also be influenced by departmental educational culture. The results additionally highlight a distinction between access to educational resources and the clinical environment in which procedural learning occurs. Although access to simulation, cadaveric training and ultrasound workshops varied between hubs, respondents frequently identified supervision as a barrier to their educational experience. The present study cannot establish whether changes to supervision would improve training outcomes; prospective evaluation would be required to examine the relationship between faculty engagement, trainer development, the clinical learning environment and educational outcomes.

Previous work has demonstrated that trainees' perceptions of the educational environment influence learner motivation, self-efficacy, engagement with training opportunities and educational performance [7]. Furthermore, validated instruments have demonstrated that trainees' perceptions can be measured reliably at departmental level and used to identify strengths and weaknesses of the clinical learning environment, supporting their role in educational quality improvement [12]. Consequently, although this study evaluated perceived training adequacy rather than objective competence, these perceptions may provide useful information about trainees' experience of the educational environment. Importantly, the present study did not rely solely on perceived training adequacy; trainee perceptions were considered alongside self-reported curriculum attainment and procedural exposure, providing complementary perspectives on the regional training programme.

Taken together, these findings support further investigation of clinical supervision within regional anaesthesia training. Whether interventions aimed at faculty engagement, trainer development or departmental educational culture would improve trainee experience or competency development cannot be determined from this cross-sectional study and would require prospective evaluation.

Variation in educational opportunities

Recent national survey data from the UK have demonstrated considerable variation in regional anaesthesia training provision, identifying differences in access to teaching, advanced techniques and departmental resources across deaneries [4-6]. Our findings extend these observations by describing variation in reported educational opportunities among respondents within a single postgraduate training programme operating under a common regional curriculum.

Despite a common regional curriculum, respondents reported variation in access to formal teaching, ultrasound workshops and cadaveric training between training hubs. Perceived adequacy of training also differed according to training hub. However, these findings should be interpreted cautiously given the small number of respondents within each hub and the absence of hub-specific eligible trainee numbers. Differential response between hubs cannot be excluded, and the observed differences should therefore not be interpreted as comparative measures of training quality between individual hubs. Perceived adequacy did not differ significantly between training stages, although the modest sample size limits conclusions regarding differences across the training pathway.

Whether increasing access to individual educational resources directly improves procedural competence cannot be determined from the present study. However, the observed differences in reported resource availability within a single training programme raise questions regarding consistency of educational opportunity and warrant further evaluation.

Although access to cadaveric courses and high-fidelity simulation was limited, these findings should be interpreted primarily as indicators of reported educational infrastructure rather than evidence of educational effectiveness. Simulation-based regional anaesthesia education has demonstrated improvements in knowledge and technical skills, although evidence regarding translation to clinical performance and patient outcomes remains more limited [13,14].

Self-reported ability, procedural exposure and competency assessment

Stage 3 respondents reported varying levels of ability to perform regional anaesthesia independently across anatomical regions, while their reported procedural experience varied considerably between individual techniques. These measures were collected at different levels - self-reported ability by anatomical region and procedural exposure by individual technique - and cannot be directly compared. They are therefore presented as complementary descriptive measures rather than evidence of a relationship between procedural volume and self-reported ability.

Self-reported ability is influenced by multiple factors, including clinical supervision, educational experiences and individual learner characteristics, and should not be considered a surrogate marker of objectively assessed procedural competence; physician self-assessment has demonstrated limited concordance with externally observed measures of competence [15]. Equally, procedural numbers alone provide an incomplete measure of proficiency and cannot account for case complexity, quality of supervision or technical performance [9]. These considerations support the continued emphasis within the RCoA Curriculum on competency-based assessment through direct observation, structured workplace-based assessment and high-quality educational feedback rather than reliance on procedural numbers or self-reported ability alone [16].

Clinical exposure provides opportunities to develop procedural experience, while supervision and complementary educational resources such as workshops, cadaveric teaching and simulation form part of the wider learning environment in which competency development occurs. The relative contribution of these components was not established by the present study. Simulation-based education can improve knowledge and technical skill acquisition, although evidence regarding transfer to clinical practice and patient-level outcomes in regional anaesthesia remains comparatively limited [14,17]. Competency assessment therefore remains crucial to distinguish self-reported ability from demonstrable clinical capability.

Educational implications

These findings identify clinical supervision, access to suitable operating lists and variation in educational opportunities as areas warranting further evaluation within regional anaesthesia training. Simulation, cadaveric teaching and structured workshops may provide complementary opportunities for deliberate practice and skill acquisition [13,14,17].

Previous national surveys have described variation in regional anaesthesia training provision, while the present study identified exploratory associations between respondents' perceived training adequacy and reported access to clinical training opportunities, suitable operating lists and supportive clinical supervision. These findings do not establish which components of the training environment should be prioritised or whether modifying them would improve competency outcomes. They provide hypotheses that could be examined in future prospective studies of procedural training.

Limitations

Several limitations should be considered when interpreting these findings. First, the survey was conducted within a single UK School of Anaesthesia and may not reflect experiences in other training programmes. The modest overall sample size (n = 64) and small numbers within individual training hubs and other subgroups limit the precision and statistical power of secondary analyses. No a priori sample-size calculation or prespecified statistical analysis plan was used, and the achieved sample size was determined by voluntary participation. Secondary inferential analyses should therefore be regarded as exploratory and hypothesis-generating rather than confirmatory. Estimates derived from particularly small groups are likely to be unstable and should not be interpreted as evidence of independent effects.

Second, the estimated response rate was 25.6%, creating a substantial risk of non-response and selection bias. Respondents may therefore differ systematically from trainees who did not participate, and the direction and magnitude of this bias cannot be determined. Consequently, the findings should not be assumed to be representative of the wider Yorkshire and Humber trainee population. Furthermore, the numbers of eligible trainees within each individual training hub were unavailable, preventing calculation of hub-specific response rates and limiting interpretation of observed differences between hubs. Reasons for non-participation were not collected, and no weighting or statistical adjustment for non-response was performed.

Third, the cross-sectional design identifies associations but cannot establish their direction or causality. In particular, associations between perceived training adequacy, supervision and access to clinical opportunities should not be interpreted as evidence that these factors independently determine training outcomes. Residual confounding and reverse associations are possible, and prospective studies would be required to examine whether modification of these aspects of the training environment improves educational or competency outcomes.

Although the questionnaire incorporated domains adapted from a previously published UK national survey and was reviewed by a regional expert before dissemination, the adapted instrument did not undergo separate formal pilot testing, cognitive interviewing, psychometric validation or assessment of reliability. Particularly, perceived training adequacy was assessed using a single study-specific Likert item, which limits the measurement validity and reproducibility of the primary outcome.

The study depended on self-reported measures of curriculum attainment, ability to perform regional anaesthesia techniques and procedural exposure, which are subject to recall bias and subjective interpretation. Procedural exposure was recorded using categorical response ranges rather than exact procedure numbers. Neither self-reported curriculum attainment, self-reported ability to perform regional anaesthesia techniques nor procedural exposure should therefore be interpreted as an objective measure of clinical competence.

## Conclusions

This regional survey identified variation in reported regional anaesthesia training experiences among respondents from a single UK postgraduate training programme. In exploratory comparisons, perceived training adequacy differed according to reported access to clinical training opportunities, suitable operating lists, and supportive clinical supervision, while reported access to educational resources varied among respondents from different training hubs. These findings are hypothesis-generating and identify clinical opportunities, supervision, and consistency of curriculum delivery as areas warranting further prospective evaluation.

Stage 3 respondents reported variable procedural exposure across individual regional anaesthesia techniques, while self-reported ability also varied across anatomical regions. Neither procedural exposure nor self-reported ability should be regarded as a surrogate marker of objectively assessed competence. These findings support continued emphasis on competency-based assessment and further evaluation of approaches to supervised clinical training, curriculum delivery and assessment of regional anaesthesia competence.

## Figures and Tables

**Figure 2:**
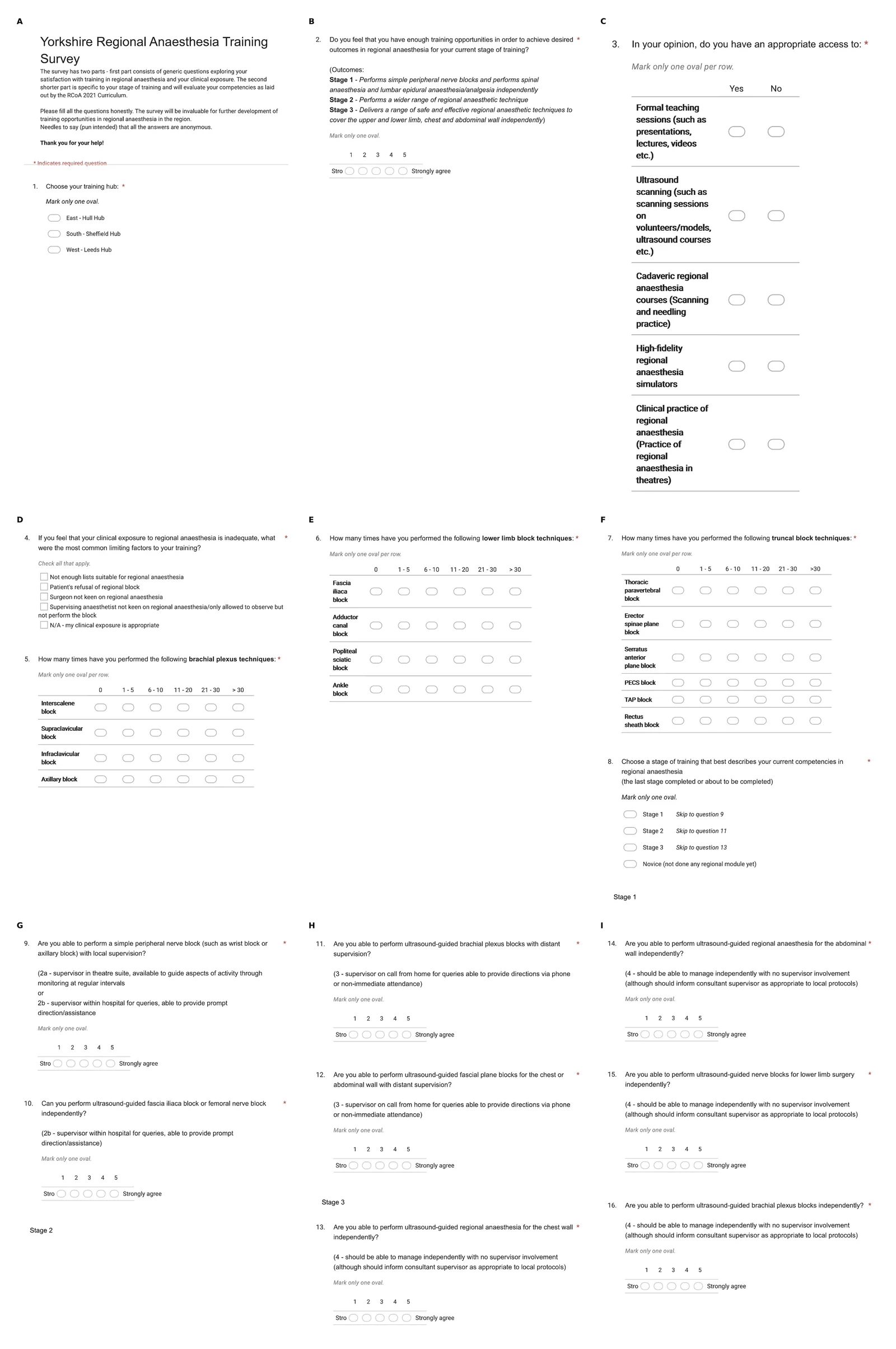
Regional Anaesthesia Training Survey Questionnaire Blank version of the electronic questionnaire distributed to anaesthesia trainees within the Yorkshire and Humber School of Anaesthesia between January and February 2024. The survey assessed trainee characteristics, access to regional anaesthesia educational resources and supervised clinical opportunities, perceived attainment of curriculum competencies, procedural exposure, self-reported ability, and perceived barriers to regional anaesthesia training. The complete questionnaire is reproduced as nine screenshots of the original electronic survey, arranged sequentially to preserve the original question wording and response options.
